# Intersecting social-ecological vulnerabilities to and lived experiences of sexually transmitted infections among Syrian refugee women in Lebanon: A qualitative study

**DOI:** 10.1371/journal.pgph.0003507

**Published:** 2024-08-08

**Authors:** Sasha Abdallah Fahme, Sara Chehab, Carmen Helen Logie, Ghina Mumtaz, Daniel Fitzgerald, Jennifer Alzos Downs, Jocelyn DeJong, Maia Sieverding

**Affiliations:** 1 Center for Global Health, Weill Cornell Medicine, New York, New York, United States of America; 2 Department of Medicine, Weill Cornell Medicine, New York, New York, United States of America; 3 Faculty of Health Sciences, Epidemiology and Population Health Department, American University of Beirut, Beirut, Lebanon; 4 Faculty of Health Sciences, Department of Health Promotion and Community Health, American University of Beirut, Beirut, Lebanon; 5 Factor-Inwentash Faculty of Social Work, University of Toronto, Toronto, Ontario, Canada; 6 Faculty of Medicine, Center for Infectious Diseases Research, American University of Beirut, Beirut, Lebanon; The University of Newcastle Australia: University of Newcastle, AUSTRALIA

## Abstract

Conflict-affected women and girls living in protracted forced displacement settings are vulnerable to sexually transmitted infections (STIs). Yet, little is known about the risk factors for and lived experiences of STIs in complex humanitarian settings, particularly in the Middle East and North Africa, where STIs have long been understudied. This qualitative study adapts the social ecological model to characterize the multi-level risks for and lived experiences of STIs among Syrian refugee women resettled in an urban refugee camp in Beirut, Lebanon. Adopting a community-based sampling strategy, community health workers, who were refugee women from the camp, recruited and conducted in-depth interviews (IDIs) with 30 adult Syrian refugee women. Data were analyzed using an interpretative phenomenological approach and thematically organized according to the levels of the social ecological model. We identified a confluence of individual, interpersonal, community-based, and societal vulnerabilities to STIs, including extreme poverty and insecurity, patriarchal gender norms, stigma, sexual exploitation and trafficking, poor healthcare accessibility, intimate partner violence, including marital rape, transactional sex, sexual harassment, social isolation, and internalized stigma. Participants described experiencing bothersome symptoms and sequelae of advanced and untreated STIs in the setting of limited access to health services and challenges with engaging their partners in STI treatment, largely due to STI stigma. These novel findings suggest dynamic, interrelated social and health disparities across all social ecological levels influencing refugee women’s sexual health, including their risk of STIs. Comprehensive, multi-sectorial interventions, which transcend traditional public health methods and which adopt a sexual well-being approach, are urgently needed to address systemic and intrapersonal violence against refugee women, examine and mitigate the burden of STIs, and ensure sexual justice and health equity in this protracted forced displacement setting.

## Introduction

Sexually transmitted infections (STIs) are understudied in protracted conflict and forced displacement settings. As the global population of forcibly displaced persons has nearly tripled over the past decade [1], displaced populations are increasingly resettling into urban environments within neighboring low- and middle-income countries (LMICs) [2–4], where structural vulnerabilities and sexual practices in the context of dynamic sexual networks may increase their risk of acquiring STIs [5–7]. Women and girls, who account for over half of the global refugee population [1], are at heightened risk of gender-based violence (GBV) and may be especially vulnerable to the transmission and complications of STIs while displaced [8–13]. Yet, there remain significant gaps in the characterization of STI risk factors and lived experiences of STIs among conflict-affected women in protracted displacement settings globally.

In the Middle East and North Africa (MENA), a region from which over half of the global displaced population originates and is resettled [14], STIs have long been underprioritized and are under-researched. Though limited data suggest that STIs may be a growing public health concern in MENA, evidence is sparse and major gaps persist [15–17]. Stigma, the criminalization of practices that elevate STI acquisition risks (e.g., sex work), and a lack of political will, all in the context of fragile health infrastructure, limit the understanding of the risk factors for and sequelae of STIs among forcibly displaced women in MENA [18–25]. The consequences of untreated STIs, which include pelvic inflammatory disease and infertility [26,27], may disproportionately impact women in MENA, as gendered stigma around infertility may exacerbate intimate partner violence (IPV), social isolation, and economic deprivation [28–30].

There is evidence that social and structural determinants of STI transmission are prevalent among forcibly displaced women in MENA [31–34], where protracted crises have led to the uprooting and urbanization of millions of people over the past three decades. These data contrast with evidence from other crisis-affected regions such as sub-Saharan Africa, where an inverse association between conflict and STI transmission has been found, possibly due to a decline in population mobility [35]. IPV, a well-described STI risk factor among women globally [36–39], early marriage, and poor accessibility to sexual health services have all been described among forcibly displaced women in MENA [31,33,40–44]. Transactional sex in the context of widespread extreme poverty and human trafficking have also been noted among Syrian refugee women [32,34,45,46], though rigorous data are lacking, given the difficulties researching these sensitive issues. Still, the mechanisms by which protracted forced displacement within urban settings influences these risk factors, and their subsequent association with STIs, remain poorly understood.

The social ecological model has been widely used to characterize and assess the multi-level, interactive risk factors for complex health problems and may be adapted to understand the impact of protracted displacement on STI risk factors and experiences in MENA. Building on the seminal work of Heise in 1998 [47], the model has been widely adapted to characterize determinants of GBV, including IPV, sexual violence as a weapon of war, sexual harassment, economic interpersonal violence, and forced marriage among diverse populations of conflict-affected and forcibly displaced women and girls, including those in Jordan, Somalia, Uganda, and Haiti [48–51]. Adaptations of the ecological model have also been used to understand GBV as a risk factor for other health problems. This model has been adopted by the World Health Organization (WHO) to conceptualize the complex interaction of individual, interpersonal, community-based, and societal risk factors for GBV on women’s health [52], and has since been applied to humanitarian contexts, where IPV, sexual-risk behavior, stigma, and condom self-efficacy may all influence women’s STI risk [53,54].

Among Syrian refugees in Lebanon, the social ecological model has been used to demonstrate that violence against refugees is systemic, with societal-level factors such as restrictive legislation, poverty, discrimination, and inaccessible health systems directly impacting intrapersonal and individual determinants of sexual and reproductive health (SRH) [31]. Importantly, Syrian refugee men are similarly exposed to this structural violence while in displacement, potentially exacerbating and driving perpetrations of violence within the household, and thus rendering intrapersonal violence in this context both structural and political [31]. Reframing IPV in the context of systemic violence against displaced men and women and applying the social ecological model to characterize the gendered pathways to and experiences of STIs may thereby render a paradigm shift in our understanding of STIs and sexual health in protracted forced displaced settings, both globally and in MENA.

This qualitative study seeks to identify and contextualize the vulnerabilities to and lived experiences of STIs among Syrian refugee women in Lebanon, applying the social ecological model to illustrate the impact of violence on STIs in this protracted forced displacement setting. Our findings address a gap in the global literature on protracted forced displacement and STIs and may be relevant to similar complex humanitarian settings to inform public health policies, humanitarian aid programming, and health resource allocation.

## Materials and methods

### Study design

This research was the qualitative component of an exploratory sequential mixed-methods study aimed at determining the prevalence, determinants, and sequelae of curable STIs among Syrian refugee women in Lebanon. The goal of the qualitative component was to explore STI vulnerabilities and characterize refugee women’s lived experiences of STI diagnoses. The data collected through this component informed the development of a survey instrument utilized in the larger study among this population.

### Study setting

The study was conducted at a community center in the Bourj-al-Barajneh Palestinian refugee camp in Beirut, Lebanon. A lower-middle-income country in MENA, Lebanon hosts the world’s highest per capita refugee population [1], and is thus an important setting to study the health impacts of protracted forced displacement. The refugee camp where the study was conducted was originally erected by the Lebanese government in 1949 to house and address the needs of Palestinian refugees under the auspices of the United Nations Relief and Works Agency [55]. Today, the camp, among the largest in Beirut, which encompasses an area of one square kilometer and has over 18,000 inhabitants, of whom 47.9% are refugees from Syria, has become part of Beirut’s sprawling, unplanned urban area [55–57]. As there is no encampment policy for Syrian refugees in Lebanon, some are self-settled in Palestinian refugee camps where rents are typically cheaper than other areas. This is particularly true following the historic economic collapse in Lebanon, which pushed over 90% of Syrian refugees into extreme poverty and poor living conditions [58]. Accordingly, the camp is characterized by slum-like conditions, including poor housing infrastructure, pest and rodent infestation, overcrowded living conditions, and an open sewage system [59]. The population dynamics of the camp, which houses Syrian, Palestinian, and Lebanese residents [55], are reflective of the diverse urban environments in which the vast majority of the global refugee population is resettled [2], and where the sexual health exposures, risks, and access to care differ vastly from those of traditional refugee camp settings, thereby making it the ideal setting to conduct this exploratory study.

Our community partner, Beit Atfal Assumoud, is a non-governmental organization (NGO) established in 1976 which provides medical care, including reproductive and mental health care, educational support, humanitarian relief, and psychosocial services to marginalized populations living in refugee camps throughout Lebanon. The community center in Bourj-al-Barajneh primarily provides psychosocial support and educational services for the surrounding community. A certified midwife is available for individual medical consultations.

### Study participants and recruitment

Study participants were adult Syrian refugee women aged between 18 and 49 years who arrived to Lebanon following the onset of the Syrian conflict in 2011 and currently reside in the Bourj-al-Barajneh refugee camp.

A community-based sampling strategy was adopted. Flyers advertising the study were posted throughout public spaces within the camp. Two female community health workers, who were also refugees residing in the camp, recruited participants from communal areas within the camp, such as marketplaces, by approaching women directly, informing them about the general objectives of the study, gauging their interest, and verifying eligibility. Community health workers also conducted door-to-door recruitment, by visiting randomly selected houses in neighborhoods within the camp known to have a high population of Syrian refugees. Women who were interested in participating provided their contact information. Community health workers then contacted women by telephone within one week, inviting them to present to the community center for an interview at a date and time that was convenient for them.

Among the thirty-five eligible women who provided their contact information, five declined to participate. The primary reason for non-participation among women who had initially expressed interest was refusal by their husbands.

### Data collection

Data were collected through individual IDIs. Written informed consent for interview participation and audio recording was obtained from all participants prior to the interviews.

Interviews were conducted in November 2022 using a semi-structured interview guide of open-ended questions and aimed to explore participants’ experiences of 1) prior STI diagnoses and treatment; 2) sexual risk practices; and, 3) displacement-related vulnerabilities to STIs. Interviews were conducted in a private room at the Beit Atfal Assumoud community center, which is located in a pedestrian alley within the camp, further promoting privacy. Interviews were conducted in Arabic, the local language. All participants consented to audio recording. Each interview lasted approximately 30 minutes. In total, 30 exploratory IDIs were conducted.

IDIs were conducted by a female refugee camp resident who underwent rigorous training in qualitative data collection methods and research ethics to serve as a community health worker in the larger study. Interviews were recorded with a digital audio recorder. Interviews were transcribed and translated into English by the community health worker. The first author, a Lebanese-American physician with experience treating reproductive tract infections among Syrian refugee women, listened to all audio recordings in the original Arabic and checked each English-language transcript for accuracy. As a form of validation, the first author iteratively reviewed and discussed the transcripts with the community health workers in real-time to elicit their perspectives on the content, specifically in the context of the patriarchal and conservative setting in which these sensitive interviews were conducted. Community health workers were trained to provide service referrals to all participants who disclosed gender-based violence and/or who disclosed or exhibited symptoms of poor mental health.

### Data analysis

An interpretive phenomenological approach informed by the social-ecological conceptual framework was utilized to analyze the qualitative data [47,60]. The first author uploaded transcripts into Dedoose V 9.0.17 (Los Angeles, CA: SocioCultural Research Consultants, LLC), which was used for the development of codes and coding. Two authors (SAF and SC) read and re-read the transcripts. Applying an inductive approach, an initial codebook was developed after reviewing two transcripts. SAF and SC independently and iteratively coded and re-coded all transcripts, applying the “constant comparison” method to assess similarities within coded excerpts across transcripts [61]. A final codebook was achieved by consensus following extensive discussions among the two authors (SAF and SC). The initial and final codebooks, illustrating the coding process adopted, are presented in a (S1 Text). The authors then independently re-coded the transcripts using the finalized codebook. Data saturation, defined as the time at which additional coding is no longer possible given the absence of new information [62], was determined to have been achieved by the authors. The coded data were then thematically organized to develop an adapted social-ecological framework which illustrates multi-level STI determinants in this protracted displacement context [47].

### Ethics statement

This study was reviewed and approved by the Institutional Review Boards of the American University of Beirut in Beirut, Lebanon (BIO-2021-0348) and Weill Cornell Medicine in New York, USA (21–07023720). All participants provided written informed consent prior to participation. All study activities conformed to the principles expressed in the Declaration of Helsinki.

## Results

**Fig 1:**
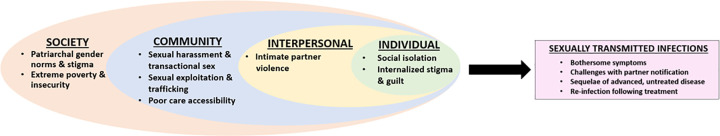
Adapted social ecological model of sexually transmitted infection determinants identified through in-depth interviews (N = 30).

**Fig 1** illustrates the experiences and drivers of sexually transmitted infections identified in this study using an adapted social ecological model. Each of these is explored descriptively below; we begin by describing lived experiences of STIs, followed by drivers of STIs spanning individual, interpersonal, community, and society levels.

### Lived experiences of sexually transmitted infections

The majority of women interviewed noted that they had been previously diagnosed with an STI, and many perceived STIs to be common in their communities. Several participants described experiencing persistent and bothersome STI symptoms, such as dyspareunia, despite receiving treatment:

“*When I first married my husband—I don’t know if it was from him or it was from me—I went to the doctor and did some tests and she told me I had given him an infection. She gave me medications at the time for me and my husband so I wouldn’t spread it to him. But could I say that I was cured? No, even until today, I haven’t been cured… When we have sex, I feel a burning type of pain. I’ve felt this every time ever since we were married, and I’ve had three children but I still feel it. I always use an intimate feminine cleansing solution, and other things too, but still whenever we have sex, I feel this burning pain.”*—IDI 7

Despite these STI symptoms, participants described challenges with partner notification. Most participants cited their husband as the source of infection; several described their husband’s refusal to receive treatment, either because of low risk perception from a lack of symptoms or because of the stigma associated with STIs. Several women described experiencing reinfection after engaging in sexual activity with their untreated partner:

“*I also suffer from this kind of infection but it’s not from me, it’s from my husband. I get treatment for it, and the doctor tells me that my husband needs to take the treatment too. I bring him the medicine home, but he refuses to take it… When I take the medicine, I feel better, I no longer have the infection, and the doctor tells me not to have sex for 15 days. My symptoms get better. But after I have sex, the symptoms come back… I even went to the Doctors Without Borders clinic and brought him the medications, but he refused to take them…He doesn’t want to make any changes. He’s not the one being harmed.”—*IDI 25

“*I have these infections*, *they’ve told me*. *I’ve been to several doctors and they’ve all told me that I have [sexually transmitted] infections*. *My husband refuses to go to a doctor*. *Definitively*. *He won’t go at all*. *He insists that he’s fine*, *that there’s nothing wrong with him*. *He has no idea that he may have an infection that he is transmitting from him to me…He refuses to get treated*. *He says ‘You expect me to go to a doctor and get treated like a woman*?*’ But if it was for something else*, *his head for example–he has migraines–he’ll see a doctor*. *But for this issue [sexually transmitted infections]*, *he won’t go*.*”—*IDI 29

This description of male partners avoiding STI care—but not other forms of healthcare—reflects the ways in which STI-specific stigma may be a barrier for men’s testing. This narrative also signals that STI mis/information regarding asymptomatic infection, and gender norms regarding STI testing (‘treated like a woman’) pose barriers for engaging male partners in testing and treatment.

Several women also described sequelae of advanced, untreated STIs in members of their community. One respondent recalled the experience of a close friend who was diagnosed with pelvic inflammatory disease (PID) after repeatedly enduring marital rape:

“*The doctors told her that her uterus is inflamed…What was causing her inflammation? She immediately knew it was because of her husband. Her husband married her by force; she never wanted to marry him. Even though they’ve had children and her daughter is grown up now, she’s about 13 years old–still, she wants to leave him because he has sex with her against her will.”—*IDI 24

Another described the experience of her cousin, a woman who presented with polyarthritis and PID, ultimately requiring a curative hysterectomy:

“*My cousin had severe infections that spread to her uterus. She had to have a surgery to remove her uterus. She got treated, she and her husband… the treatment lasted a while, maybe 5 or 6 months. She used to feel pain in the joints of her legs and hands. She wasn’t able to walk from the pain. So she went to a doctor, and he told her that she had [sexually transmitted] infections that were impacting her whole body.”—*IDI 21

In sum, refugee women participants often experienced persistent and bothersome STI symptoms yet were largely unable to engage male partners in testing and treatment due to the confluence of gender norms regarding men’s healthcare seeking, STI stigma, and low risk perception. This may be exacerbated by gender-based violence and inequitable relationship dynamics.

### Individual-level factors

#### Social isolation

Women’s experiences in displacement were characterized by family separation, mobility restrictions–both self- and partner-imposed–and consequent social isolation. Social isolation in Lebanon was contrasted against women’s experiences in pre-conflict Syria, during which time many recalled enjoying rich social lives with extended family support:

“*It was better in Syria. Life was sweeter…A person was living in his own home and knew his family, his neighbors. [In Lebanon], you don’t know anybody, you don’t visit anybody. You just stay put in your home.”* -IDI 14

“*I don’t have any friends or acquaintances who visit me*. *I’m alone*. *After I got married*, *I feel like I’ve withdrawn from society*.*”—*IDI 11

The social isolation many women described experiencing in Lebanon was often exacerbated by physical confinement in their homes, either due to safety concerns and/or because their husbands did not permit them to leave. A participant described such safety concerns in the host country: *“It is not safe here [in Lebanon]*. *Here*, *a woman can leave the house and not know if she will return*.*”—*IDI 4

Another discussed fear of violence and unfamiliar settings: *“I’m afraid*. *I’m afraid of all the people*. *Sometimes*, *I don’t like walking alone outside the home*. *I’m scared someone will kidnap me*. *There are neighborhoods [in the camp] close to where I live that I don’t like to go to*. *Unfamiliar places*, *I avoid*.*”*- IDI 11

Indeed, several women likened their displacement in Lebanon to being imprisoned:

“*[The men] had stopped us from going outside the home because they were scared that we might clash with the neighbors, and because it wasn’t safe. Imagine that just stepping foot onto the building stairwell felt like we were out on an excursion!”—*IDI 20

#### Internalized stigma and guilt

In the context of social isolation and harsh living conditions, many participants exhibited symptoms of depression, including guilt. Several women interviewed described experiencing sexual violence, and most recounted the trauma of not being able to discuss their experiences with others, including other women, due to internalized sexual violence stigma:

“*I fell into a depression…I stayed silent, silent, silent for I swear to God, more than two months. I wouldn’t see any one and no one would see me. If anyone asked me why I wasn’t going out, I would tell them that I was busy at home. Only God knew what was in my heart. You can’t talk about these things to a sister, or a neighbor, or anyone. They would blame me; they would think that I must have said or done something to make him think that he could proposition me. But I know myself. I didn’t say or do anything to him. He took advantage of me all on his own.”—*IDI 7

Participants additionally suffered from feelings of guilt related to the challenging economic circumstances they and their children face in Lebanon:

“*Sometimes [my daughter] goes without food. She’s just one year old. What has she done to deserve this? I blame myself. Sometimes, I think to myself, if I can’t even feed her or make sure she’s healthy, how could I have brought her into this world? … It’s hard and sometimes I wish I never had children… Every time I see her, I feel guilty.”—*IDI 11

Together the convergence of social isolation and fear, with internalized stigma and guilt, could harm mental health, in turn shaping participants’ lived experiences of sexual health and sexual wellbeing, including STIs.

### Interpersonal-level factors

#### Intimate partner violence

Participants’ sexual wellbeing and autonomy are further constructed by their lived experiences of emotional, physical, and sexual IPV, which many women interviewed described being committed by their spouses:

“*Sometimes if I am upset or feeling overwhelmed, and don’t want [to have sex] with my husband, he’ll force me. I hate him for it. Why isn’t he considerate of my wishes?… Like today, for example, he wanted to [have sex], and I told him that I didn’t want to, I wanted to rest. So he said ‘No. I want to. You do not have any say in it. It’s not up to you. You are forced to do this. You are a woman, you are only here to serve. The woman should obey her husband. The woman who does not obey her husband will be punished.’”*—IDI 29

Another participant narrative described verbal IPV: *“Even me*, *my husband speaks to me cruelly–his words hurt me*. *They feel hurtful*. *As a woman*, *I feel hurt… Thankfully*, *he doesn’t beat me*. *Only words*. *But sometimes words cause more pain than fists*.*”—*IDI 22

Other women recounted similar instances of IPV, including marital rape and physical violence, that they witnessed perpetrated against other Syrian refugee women, including their mothers, sisters, cousins, friends, and neighbors:

“*My sister gets beaten by her husband…She gets beaten so severely, so severely that even the Lebanese and Palestinian neighbors had to intervene to physically pick him up off of her. He beats her and her children… If you saw him outside the house, you would think that he is a calm and wise man. But when it comes to his wife and daughters, he’s the opposite. He keeps hitting, and beating, and kicking them to such an extent that he might kill them if someone didn’t stop him. You can see in his heart an anger that’s been there for years.”—*IDI 24

Many described that men became violent when their wives declined to engage in sexual activity, sometimes in the context of forced or early marriage:

“*There is man who constantly beats his wife. He tells her he is not satisfied unless he is beating her… Her body is covered in bruises. He beats her properly and when he’s done, he rapes her… This woman I’m telling you about got married at 16. She tells me that the [physical and sexual abuse] is normal—that it’s his right–and that she is used to it…Maybe if she were a bit older she would have defended herself, or told someone that if he kept up this behavior she wouldn’t stay with him. She’s not obligated to live a life like this.”*—IDI 30

“*Our neighbor was living just below us on the fifth floor*. *Whenever her husband wanted to have sex…he would force her to have sex with him…He would beat her and insult her every time he wanted to have sex and every time*, *the entire neighborhood would hear it so he was exposing himself for what he really was*, *all for sex*.*”—*IDI 15

One interviewee who worked at a hair salon recalled an incident involving one of her clients:

“*One time a woman came to the salon who was forced to marry an older man. He abused her very much. One time they fought and he cut off all of her hair, down to the scalp.”*—IDI 4

These narratives reflect multiple forms of IPV faced by Syrian refugee women, including sexual, verbal, physical violence, and how this relationship violence and lower sexual relationship power may be exacerbated among younger women who experienced early and forced marriage.

### Community-level factors

#### Sexual harassment and transactional sex

Beyond the context of intrapersonal and intimate partner relationships, many women described sexual harassment and propositioning in community spaces as both stressful and commonplace, particularly by shopkeepers to whom women may be financially indebted. One woman described:

*“Once, I went to the grocery store to buy some vegetables. The shopkeeper gave me vegetables. I told him I would pay him as soon as my husband received his salary and he agreed. About four or five days later, I needed some vegetables and returned to the store…I know this man. I’ve lived a lifetime in this camp, not just a day or two. So the second time I visited the shop I brought back the vegetables, and the third time I returned to pay him the money. I told him that thank God things were a bit easier at home and that I’d brought him the money. He held my hand and said: “I don’t want the money. I want you. Whatever vegetables you need, the entire store is yours.” I threw the money at him and left.”—*IDI 7

Indeed, several women noted that transactional sex in exchange for essential goods—sometimes termed “survival sex” in the literature—was a necessary means of addressing debt or responding to food insecurity, particularly among women with young children and those whose husbands are unemployed:

“*I’ve heard a story very recently of a woman who owes money to a shop owner. The shop owner told her she needed to pay her debts and she told him she wasn’t able. She asked him if he would give her more time to pay and he told her he wouldn’t wait any longer. He propositioned her to have sex instead…I’ve heard many stories of men who exploit women in this way if she is unable to pay her debts. They ask her for sex.”—*IDI 9

“*I’ve heard [shop owners] say things like*: *‘I’ll give you everything for free’ or ‘Take what you want and in return give me…’”—*IDI 11

One woman asserted that sexual harassment by employers was a major deterrent to seeking employment:

“*I tried to work cleaning people’s houses but I found it very difficult. One time, they arranged a house for me to clean, but I felt like the situation wasn’t right so I left and I’ve never turned back… [I heard about] a man who tried to sexually assault [a Syrian refugee woman]. He said to her: “You are worth less than my shoes, you have to do what I say” This kind of filthy language. This is why I don’t want to work outside. I’m telling you that I wanted to work, but I felt that something wasn’t right so I immediately backed out.”—*IDI 15

These narratives signal contexts of sexual harassment and transactional sex for survival in local shops, as well as in places of employment, that reduced sexual safety and security. Such experiences are reflective of gendered power dynamics by which the control of essential resources, such as food, is retained by men who may be emboldened to demean or deprive vulnerable women of goods as a means of asserting their authority within community spaces.

#### Sexual exploitation and trafficking

The deliberate exploitation of vulnerable women and girls by men who posed as humanitarian aid workers throughout the refugee camp was described in detail by several participants:

“*There are people who take advantage of women during Ramadan. They bring a woman food parcels in order to flirt with her, to get close to her, to go inside her house and sit with her… I’m 100% sure that I saw them [having sex]. Imagine a woman who has only one daughter and her husband is at work all day, and when he comes home at night he brings home 3 or 4 sandwiches that are meant to feed the entire family for a full day. And the next day, same thing. Even when her husband isn’t working, he can call a friend who can send him over some food. But a woman…try to imagine: she wants a cup of milk, and she’s always complaining that she doesn’t have enough to buy a cup of milk. Well of course, she’s complaining because she wants [the men] to hear her–evil men with bad intentions, barbaric, the ones who want to take advantage of women. I saw them bringing her things. This is exploitation.”—*IDI 24

“*He was in the camp distributing humanitarian aid to people*. *He said that women were coming to him wearing only an abaya [traditional covering] with nothing on underneath*, *just so that he would give them aid… he was either exploiting them or forced them [to have sex]*. *And he was married and had children–he wasn’t in need [of sex]*.*”—*IDI 16

“*There was a man in the marketplace registering names to distribute aid parcels…he told me that the parcels were in his house and that if I wanted to pick up a parcel I could go to his house… I said no*, *let me find someone to go with me to the house…he said ‘No*, *go by yourself and I will take you home after*.*’ So I told him that I don’t want the parcel anymore*. *Of course*, *I had heard about this man before*, *but I didn’t know him personally*. *I told him I no longer wanted these things*, *and now I can no longer walk by the marketplace where he stands… Whenever I tell anyone about this story*, *they say it’s true*, *and that they faced the same thing…After that*, *he started hanging around where I live*, *distributing goods from there*. *He saw me and told me that me that if I had only registered my name with him before*, *I would have received assistance*. *But I told him I don’t want to register*.*”—*IDI 25

These narratives reflect how poverty and food insecurity elevate refugee women’s exposure to sexual exploitation, again in the context of gender disparities in resource control. Another woman described the attempted trafficking and forced sex work of young girls by an individual who claimed to operate a women’s rights organization for girls who were married before the age of 18:

*“There were men kidnapping newlywed girls who were just sixteen or seventeen years old… Many of the girls who this happened to remained silent, but one of them could not remain silent about what happened to her. One time, we were gathered and this girl approached me to tell me: ‘This man who registered girls who married before the legal age [of 18 years] said he wants to do awareness sessions for us.’ He announced this in front of her parents, that they hold awareness sessions and that since she was married before turning 18, she was qualified to participate in these sessions, and that she would earn a percentage of the money that they raised for participating in these sessions, and that every now and then they would give her a food parcel because she is under the legal age for marriage. About a week later, he called her to attend the first session, but the center was not a proper center–it was an empty house with chairs. She said: ‘At first, we sat, and it was a normal session. The man was explaining things and two girls were participating. But then, the man began to take each girl aside one by one and ask them if they could come see him individually at a certain day and time and that no one would know because they are participating in sessions and that he would give them a good amount of money. One of the girls who was shy told him not to speak to her that way again.’ It turned out that the center is actually a front for [sex]. There are a few men who are involved.”—*IDI 30

These narratives suggests that young age at marriage, combined with poverty, may exacerbate risks of sexual exploitation among women refugees by men pretending to offer humanitarian assistance.

#### Poor healthcare accessibility

The multi-level factors shaping women’s lived sexual health experiences are further shaped by poor healthcare accessibility. Many women reported being unable to access medical care during forced displacement in Lebanon, largely due to the prohibitively expensive costs in the setting of a fragmented and privatized health system. Some women described resorting to giving birth at home as they were unable to afford the cost of a hospital delivery:

“*In Syria, all pregnancy-related care and even delivery was free. Even if I went to a private doctor, it didn’t cost me a fraction of what it costs here. When I came here, I was seven months pregnant. We asked how much delivery in a hospital would cost, and they told me it would be one million Lebanese Lira. One million LBP for us is a lot of money. So we asked around for a midwife, and she delivered my baby at home, in the home of a Lebanese family. I couldn’t pay a million LBP. We paid her 200,000 LBP.”*- IDI 18

“*Here [in Lebanon]*, *we are all living together in one small room*. *I gave birth to two daughters in the small room*.*”—*IDI 16

The majority of participants additionally described no longer being able to afford medical consultations with a physician following Lebanon’s economic crisis. Some with previously diagnosed STIs instead relied upon pharmacies when they experience recurrent symptoms, while others are not able to afford treatment altogether when experiencing sexual and/or reproductive tract infections:

“*I used to go to the pharmacy to get medicines because if you go to the doctor, you have to pay 200,000 or 300,000 LBP at least. So whether it’s my daughter or my son or me who’s sick, I go to the pharmacy instead to get medications… I get these infections every now and then. I go to a pharmacy to take medicine and they help. A couple of days ago I started to have burning when I urinated. I got pills dosed 500 mg from the pharmacy but my symptoms didn’t go away. I went back to the pharmacist and told him about my situation so he gave me a different medicine. And I took it—I didn’t see a doctor.”—*IDI 26

“*My mother gets sick with these infections*. *She tells me about these infections now because I’m 18 years old*. *She talks to me about it sometimes and tells me that she has infections that may be sexually transmitted by my father*. *She doesn’t explain that much to me beyond that*. *Sometimes she goes to the doctor and does tests*, *but sometimes she doesn’t and just waits for it to get better without treatment*.*”—*IDI 10

In sum both reproductive and sexual health care was described as unaffordable, with the result that many refugee women participants were unable to access SRH care, including for pregnancy and childbirth as well as for reproductive tract infections.

### Society-level factors

#### Patriarchal gender roles and stigma

The individual-, intrapersonal-, and community-level determinants are experienced in the context of overarching societal factors, notably including patriarchal values and systems in which women are devalued. Several participants felt that such customs are upheld to normalize violence against women, in particular marital rape, which women noted was inaccurately justified as a married woman’s ‘obligation’:

“*Here, they think marriage means that a woman must do everything a man wants. He married her [for sex] and many times a woman may not want to [have sex], she could be sick for example or have her own reasons [for not wanting to have sex], but it doesn’t matter… Many relatives who are very close to me have experienced [marital rape] and it’s had a big effect on them… And when she confides in anyone from our community, they would all simply tell her that it is a woman’s duty to have sex with her husband. It is one of the woman’s duties. No one would help her. They would tell her that she is obligated to have sex with her husband, that she is forced to [have sex] because she is married, and her husband is entitled [to sex].”—*IDI 27

This above narrative reflects the belief that married women do not have the right to refuse sex from their husbands and this is reinforced within gender norms and values. Indeed, discussing marital rape could also be stigmatized. For instance, stigmatization of sexual violence and marital rape were cited by respondents as major barriers to disclosure and care-seeking: *“One of our relatives is raped by her husband*, *but she doesn’t talk about it*, *she’s not able to—do you understand what I mean*?*”—*IDI 10

Rigid patriarchal gender roles and legislation which favor paternal custodial rights were also cited by several participants who described enduring IPV for the sake of remaining with their children. One woman described suffering from marital rape because she did not have anywhere else to live and was fearful of losing custody of her children: *“I do not have anywhere else to go*. *I do not have a safe place*, *like my mother’s home for example*, *where I can go*. *I am forced to stay and spend 24 hours a day with him for the sake of my children*.*”—*IDI 29

In another example, a participant noted: *“If a man beats his wife*, *she could leave him and go back to her parents’ house*. *But if she decides to stay with him*, *she’s staying for her children and not for her husband*. *She’s enduring this abuse for her children*.*”—*IDI 21

In addition to experiencing gender-based stigma embedded in paternal custodial systems, women described fears of negative social repercussions such as ostracization for seeking separation or divorce from a violent intimate partner:

“*I feel, in our culture, [divorce] is considered shameful, but in my opinion, it is not so. It is not shameful. But if a woman wants a divorce, everyone starts intervening. For example, my husband and his brother and others intervened with my sister, saying that it was shameful [for her to leave him], and that she should let it go, and that people will start saying that after they fled [Syria], she became promiscuous. Fine! Let us be promiscuous! Is it better for her to die than to be promiscuous?”—*IDI 24

This narrative not only signals divorce-related stigma, but also the ways in which women’s sexual agency could be stigmatized when wanting to end a marriage.

Participants also reported stigma toward transactional sex and sex work. Many participants paradoxically displayed similar contempt for their compatriots who experience sexual harassment or exploitation while displaced in Lebanon. Though many women shared stories of sexual harassment and propositioning, none disclosed personally engaging in transactional sex work. Several participants noted that the high stigma toward transactional sex and sex work reduced the likelihood of disclosing one was engaging in transactional sex. Indeed, transactional sex was characterized by many as “a violation of one’s honor” and regarded as highly shameful from both a cultural and Islamic perspective:

“*We’ve heard many stories of women who sold their honor just to secure their needs. This is something wrong. This is the biggest mistake a woman could make. We see them on the streets. It’s wrong. Other women, even if they’re just selling gum on the street to be able to support her children, haven’t forsaken their honor.”—*IDI 16

Together these narratives suggest that larger social contexts of patriarchal inequitable gender norms are used to justify IPV, including marital rape. This gender-based stigma co-occurs with other gendered forms of stigma, including divorce-related stigma and transactional sex/sex work-related stigma.

#### Extreme poverty and insecurity

Similarly, and in the context of the historic economic collapse in Lebanon, extreme poverty in the slum community where participants reside may further exacerbate the identified social-ecological factors, with many participants describing being unable to meet essential needs, such as food, water, and shelter:

“*I was pregnant, and all I could afford to eat was a piece of bread that I would heat on the gas stove. We were deprived. The rent was $250 and we couldn’t afford a generator, so we were sitting in dark. We had a tiny gas stove that we would use just to heat some bread.”—*IDI 15

“*You have to take food out of your children’s mouths just to secure rent*, *so that the landlord doesn’t tell you ‘If you don’t like it*, *get out and find another house*.*’”—*IDI 1

Several participants explained that women were typically responsible for securing essential household needs in Lebanon, leading to distress and in some cases depression in the setting of hyperinflation:

“*We never had to worry about anything [in Syria]. This was before the war. But currently, the man works and the woman has to handle all the household expenses with the allowance he gives her. So here [in Lebanon], maybe I’ve grown far beyond my age, from the very day I arrived. I am responsible for everything. The man works, he gives you an allowance, and you have to figure out how to make it work.”—*IDI 6

In the context of these dire economic conditions, some women resorted to begging or child labour to secure essential needs. One woman feared that her children could be exploited while begging in the street, but noted that she had few other alternatives:

“*My children work on the streets. They sell water and biscuits…They are the ones who bring home money. With the money they make, I buy them bread. Sometimes I buy them rice to cook for them…I worry that people will take advantage of them and such. By nightfall, my mind is about to burst with worry. But what can I do? If they stay at home, they will complain all day that they are hungry. At least, when they go out into the street, a person may buy them a sandwich.”—*IDI 29

Indeed, the severity of the Lebanese economic crisis was likened by some to the war in Syria: *“We faced a crisis of war in Syria*, *then we came here and found another crisis*: *the dollar*.*”—*IDI 26

Many women described economic stressors and food insecurity as major sources of household tensions and IPV:

“*My sister’s husband would go four five months without work*. *They were living on the sixth floor and my parents were on the third*. *She had five or six kids*, *and she would have to get food to feed them from my parents*. *She would beg them for food*, *even though they’re her parents but still*, *she was begging them*, *in order to feed her children*. *And then she would go home and her husband would beat her saying ‘Who told you to bring home food*?*’ One time I was at her house and she had marks all over her body from the water hose he used to beat her*. *She was wearing pajamas to cover herself and I asked her why she was wearing them because it was so hot that day*. *As soon as she took off her pajamas*, *I saw her whole body was black and blue with bruises*. *All because she brought food up from her mother’s house*. *If she didn’t go down to get food*, *how could her children eat*?*”*—IDI 7

These participant narratives signal that profound economic insecurity elevate refugee women’s stress and risks of IPV, alongside refugee children’s risks of hunger and sexual exploitation.

## Discussion

Our results indicate that a confluence of individual, interpersonal, community-based, and societal determinants may cluster together to influence Syrian refugee women’s experiences of STIs and sexual health at large in a protracted forced displacement setting. In a region where STIs have been historically neglected and assumed to be negligible despite a paucity of rigorous epidemiologic data, these findings call for greater surveillance to screen for, diagnose, and treat STIs in this vulnerable population. These findings also reflect the importance of addressing both mental health and sexual wellbeing among refugee women, which is a multifaceted construct that includes sexual health (including STI and sexual violence prevention), sexual justice (human rights and equitable SRH experiences), sexual wellbeing (including sexual respect, sexual safety and security, self-determination in one’s sexual life), and sexual pleasure [63].

Individual-level risk factors identified in this study, including internalized stigma, social isolation and depression have been shown to be significantly associated with STIs in diverse high-resource and LMIC settings, potentially through increased sexual risk practices and hesitancy to seek care [64–67]. Among refugee young women in Uganda, for instance, lower adolescent SRH stigma was associated with both increased STI services awareness and STI testing uptake [54]. While depression is prevalent among refugee populations [68–70], including among Syrian refugees [71–73], and associated with social isolation in these populations [68,74,75], the association with STIs is understudied. However, a recent study measuring the syndemic impact of depression, war trauma, stress, and IPV on STI risk among a similar, though non-displaced, post-conflict population of adults in Liberia, found an association between depression and STIs, potentially mediated by IPV [67]. Another recent LMIC study with refugee youth reported an interaction between poverty and recent sexual and gender-based violence on reduced HIV prevention motivation [76]. Other non-conflict setting studies have noted that depression is associated with lower odds of HIV testing, suggesting poor mental health is a barrier to realizing optimal sexual health [77]. Notably, multidisciplinary interventions addressing both IPV and mental health have been shown to be feasible among refugee women in resource-limited settings [78]. We argue that such an integrative approach is necessary in the Syrian refugee context as well, as the vulnerabilities identified in this study may have synergistic adverse effects on both the mental and sexual health of women in this community.

Our study findings suggest that social isolation and self-censorship due to internalized sexual violence stigma may contribute to depressive symptoms among urbanized Syrian refugee women in Lebanon, potentially influencing STI experiences through healthcare-seeking behaviors. Social isolation and internalized sexual violence stigma were also found to be significant barriers to STI prevention and management among refugee girls and women in Uganda [79]. Several qualitative studies with refugee women and youth have demonstrated that shame, guilt, and low self-esteem may contribute to women and girls’ self-isolation and reluctance to disclose violence or visit healthcare facilities, particularly in their own communities [79,80]. As Syrian refugee women in Lebanon experience mobility restrictions [31,41], reluctance to seek resources in their own communities due to stigma may preclude them from accessing care altogether.

At the interpersonal level, potentially pervasive IPV among urban Syrian refugee women in Lebanon may be an important STI determinant in this setting. A bi-directional association has been described by which IPV, through sexual violence, coercion, and diminished capacity for sexual and condom negotiation, promotes STI transmission [81,82], while partner notification and patient-delivered partner therapy following an STI diagnosis may instigate or exacerbate IPV [83–85]. Several women in this study recounted challenges with partner notification and treatment, suggesting a potentially similar relationship among this population. Armed conflict and displacement-related factors may confer intersectional vulnerabilities to both IPV and STIs among forcibly displaced women [31,33,51]. A 2014 cross-sectional study of Syrian refugee women in Lebanon demonstrated that IPV was significantly associated with reproductive tract infection symptomatology, though did not incorporate etiologic testing for STIs [43]. Our results indicate that sexual violence, and particularly marital rape, may be a significant concern and a potential driver of STIs within this population.

Other interpersonal-level factors such as sexual harassment, transactional sex, exploitation, and trafficking recounted by study participants should be examined using an intersectional lens that contextualizes these risk factors in a setting of extreme poverty and insecurity, patriarchal hegemony, and anti-refugee racism. For instance, a 400% increase in food costs since 2019 in Lebanon have led to a near doubling of food insecurity among Syrian refugees [86]. While food insecurity has not been studied as an independent predictor of sexual-risk behavior or STIs in this context, the UN Refugee Agency (UNHCR) has implicated growing insecurity as a driver of “crisis- and emergency-level coping strategies” among Syrian refugees in Lebanon [87]. Sexual exploitation and human trafficking have similarly been anecdotally reported by local and international NGOs and in governmental reports, though the prevalence of these human rights abuses among Syrian refugee women and girls remains unknown [32,88].

Notably, there are inconsistent reports of transactional sex as a coping mechanism among Syrian refugee women in Lebanon, with the practice frequently described among key informants but rarely among women themselves [89]. In our study, women similarly shared stigmatizing views of transactional sex, and personal experiences of sexual propositioning, but notably did not disclose engaging in such acts themselves. Our findings indicate that Syrian refugee women are responsible for overseeing household expenses to secure shelter, food, electricity, and children’s tuition, suggesting that they may be at higher risk for coping strategies such as transactional sex, and may be disproportionately affected by trafficking attempts. The association between food and water insecurity and sex work has recently been demonstrated among young adult refugees in Uganda, where food and water insecurity conferred a three-fold greater risk of engaging in sex work [90]. We will quantitatively examine the relationship between food insecurity, negative coping strategies, and STIs in the larger mixed-methods study to better understand STI determinants in this sample of urban Syrian refugee women.

Sex work stigma and the criminalization of sex work in Lebanon may have discouraged participants from speaking freely on issues related to sex work and exploitation [24,91]. We found stigma to be common among participants, some of whom shamed women in their communities for resorting to such “violations of honor”. Such sentiments are reflective of wider, society-level values and gender norms which decry women’s sexuality [92], and are likely compounded by conditions of extreme poverty. For instance, a multi-national survey of men across four MENA countries found that belief in gender equity correlated with socioeconomic status, and was more common among wealthier men, those who had attained a higher level of education, and those with educated mothers [93]. It is therefore plausible that such beliefs are held too by women in this setting, who may regard the upholding of their “honor” as a form of pride and resilience in the face of extreme economic marginalization. Still, further research is needed on optimal methodologies to measure transactional sex accurately in contexts of sex work stigma and determine its association with sexual health outcomes, including risk of STI transmission.

Characterizing STI risk factors among Syrian refugee women in Lebanon is particularly significant given this population’s severely constrained access to health services [24,33,94]. Unlike the free primary and reproductive health services available in pre-conflict Syria, Lebanon’s highly privatized and fragmented health system prioritizes tertiary and specialty care [33,94]. Prior studies have cited high costs, fear of mistreatment, and a paucity of female clinicians as barriers to healthcare accessibility among Syrian refugees in Lebanon [24,31–33,95], with informal health systems staffed by undocumented Syrian healthcare workers operating in parallel to fill these gaps [96,97]. Our results are aligned with these findings, suggesting that STI stigma, particularly among men, may be a major obstacle to receiving care, and that women in this community may be increasingly reliant on pharmacies due to prohibitive out-of-pocket healthcare costs. Pharmacies may therefore be a pragmatic setting to both screen for sexual risk practices among Syrian refugee women seeking care for STI symptoms, as well as engage their partners, who as our findings suggest, may be difficult to engage and retain in care. Further research on pharmacy-based, social-ecological approaches to assessing STI risks and sexual health needs in the context of violence, poverty, and intra-relationship STI disclosure dynamics among this population are needed.

This study responds to a global gap in the literature on refugee sexual health in protracted forced displacement settings. As two-thirds of the global refugee population is displaced for five or more years in LMICs [14], it is imperative to characterize and address refugee women’s unmet sexual health needs in these settings. In Lebanon, where an estimated one in four persons is a refugee [1], a growing body of literature has called attention to Syrian refugee women’s SRH needs, including reproductive tract infection symptoms, poor knowledge of and access to contraceptives and family planning, and insufficient antenatal, postnatal, and labour and delivery care coverage [33,43,98–100]. Studies have further demonstrated maternal health disparities among Syrian refugee women in Lebanon, including disproportionate rate of cesarean sections and a high burden of obstetric violence during labour and delivery [101,102]. These disparities have been further exacerbated in the context of compounding crises, including protracted political instability, economic collapse, and the Covid-19 pandemic [103]. Yet, sexual health needs, including STIs, beyond those related to pregnancy and family planning remain under-characterized in this population, reflecting the prioritization of other, potentially less stigmatizing, health needs. Sexual health is often overlooked in emergency settings, despite the implementation of the minimal initial services package (MISP), in which the Inter-Agency Working Group on Reproductive Health in Crises outlines the basic interventions to maintain women’s SRH in the acute phase of a humanitarian crisis [104]. Yet, the protracted nature of forced displacement imposed by the Syrian conflict–now in its thirteenth year–necessitates a more comprehensive examination of refugees’ sexual health that extends beyond reproductive health and the MISP objectives [105]. The findings of this formative study provide a framework for contextualizing sexual health in this population and represent the first step towards responding to these unmet needs.

Our findings additionally have important practice implications for clinicians addressing IPV and STIs in this context. Many of the social-ecological factors described in this study, including extreme poverty, insecurity, and poor healthcare accessibility similarly impact both Syrian refugee men and women in Lebanon, and may provoke and/or worsen IPV in this context. Our results are aligned with arguments made by Yasmine and Moughalian, who conceptualize IPV among Syrian refugee women in Lebanon as a form of social injustice enabled by discriminatory systems and human rights abuses including labor force restrictions, limited educational opportunities, shelter insecurity, inaccessible health and legal systems, and pervasive anti-refugee xenophobia, perpetrated against Syrian refugee men and women and facilitating conditions conducive to violence within households [31]. While this institutionalized violence does not justify IPV, it must necessarily be addressed when designing IPV, mental health, and STI prevention campaigns in this context, warranting a multi-sector, comprehensive approach that expands beyond traditional public health efforts.

Our study has several limitations. Firstly, as above, women often provided perceptions rather than firsthand accounts of certain sensitive issues such as transactional sex, though many participants shared personal experiences of other stigmatized topics such as marital rape and sexual propositioning. Secondly, study participants were recruited from a single urban slum community in Beirut with potentially unique exposures and thus, the vulnerabilities identified may not apply to Syrian refugee women living in other, and particularly rural, settings. Additionally, though participants offered accounts of their experiences with STIs, we did not conduct STI testing, which is beyond the scope of this formative research. Finally, the study population was limited to cisgender Syrian refugee women and thus, these findings may not apply to transgender and gender non-conforming individuals, who experience distinct sexual health vulnerabilities in Lebanon [106]. Further research which additionally examines STI prevalence is needed among larger populations of Syrian refugee women across diverse settings to validate these findings and better characterize sexual health needs. Future studies should also engage men in this community, who may represent the primary mode of STI transmission. Despite these limitations, there are valuable insights on sexual health determinants to be gained by studying Syrian refugee women in Lebanon, a refugee-dense LMIC with compounding protracted humanitarian crises, which can be applied to other resource-limited settings of protracted conflict and displacement, particularly in conservative settings.

This study also has a number of strengths. To our knowledge, this is the first exploratory study of STI vulnerabilities and lived experiences among Syrian refugee women globally, and the first to apply and adapt the social ecological model to characterize and contextualize STI experiences in MENA, where STIs have been long understudied. Utilizing a community-based sampling strategy, we trained refugee women to recruit and enroll participants who may not otherwise be engaged in care, and thus may not be represented in studies typically conducted among convenience samples of clinic attendees or NGO beneficiaries. Training a refugee woman from this community to conduct the in-depth interviews, a strategy supported by a growing body of research demonstrating the benefits of refugee-led research and health interventions [107,108], may have also encouraged participants to share more freely and contributed to the richness of our data.

## Conclusions

Urban-dwelling Syrian refugee women’s experiences of STIs in Lebanon are characterized by intersecting forms of stigma, shame, and challenges related to partner notification, potentially leading to complications of advanced and untreated disease, and adversely impacting mental health. Our findings document interconnected social (societal and intrapersonal violence, poverty), and health (reproductive tract infections, STIs) disparities among this displaced population that are driven by multi-level, dynamic factors spanning social ecological levels. Sexual health and wellbeing data, including STI diagnostic data, are needed to delineate the mechanisms by which the individual, interpersonal, community-based, and societal factors identified in this study may shape sexual health outcomes, including increased STI acquisition risks and reduced access to care. A sexual wellbeing approach offers the potential to not only address refugee women’s sexual health through reducing exposure to STIs and IPV, but to advance sexual justice, sexual safety, and self-determination, in turn advancing health equity.

## Supporting information

S1 Checklist Inclusivity in global research. (DOCX)

S1 Text The initial and final coding trees. Within each coding tree, parent codes are bolded and related child codes are indented directly underneath.
(DOCX)
