# Supplementary material for: Intersecting social-ecological vulnerabilities to and lived experiences of sexually transmitted infections among Syrian refugee women in Lebanon: A qualitative study
Source: PLOS Glob Public Health. 2024 Aug 8;4(8):e0003507. doi: 10.1371/journal.pgph.0003507 (PMC11309427; doi:10.1371/journal.pgph.0003507)
Supplement: S1 Text — Within each coding tree, parent codes are bolded and related child codes are indented directly underneath. (DOCX) [file pgph.0003507.s002.docx]

**Initial Code Tree**

**Women’s sexual and reproductive health**

- Pregnancy
  - Delivering in Lebanon
  - Difficulty getting pregnant
  - Miscarrying during war
  - Pregnancy during war
    - Hospitals not safe
- Rape
- STIs
  - Conflating RTIs or UTIs with STIs
  - Discussing STIs with other women
    - Not discussing STIs
  - Getting treatment for STIs
    - Getting treatment at pharmacy
    - Getting treatment by doctors /clinic
  - Husband with STI
  - Knowledge about STIs
    - No Knowledge about STIs
  - Purposefully transmitting STIs
  - STI symptoms
  - STIs leading to infertility/stillbirth
  - Women with STI

**Disintegration of social structures**

- Children impacted by the move to Lebanon
- Dependence on men for moral support
- Disintegration of the family unit/structure
  - Women by themselves or with their children alone

**Syria before the war**

- Syria changed
  - Nothing to return to
- Syrian War
  - Destruction
  - No communication with people in Syria

**Wanting to return to Syria**

**Men during war**

- Men forced to join group
- Men imprisoned/ military so cannot return
- Traveled for work
  - Husband already in Lebanon

**Women keeping to themselves and not interacting with society**

**Discrimination in Lebanon**

**Restrictions on women's mobility and freedom**

- Dependence on men for safety
- Difficult journey
- Forced marriage
- Freedom of movement
  - Freedom of movement for children
  - Men go outside women stay inside
  - Movement in Lebanon
    - Lebanon is like a prison
    - Men restrict women's movement in Lebanon
  - Women have less freedom of movement

**Reversal of gender roles**

- Men losing masculinity
- Women responsible for themselves/family in Lebanon
- Women responsible for themselves/family in Syria

**Economic dependence on men**

**Early marriage**

**Intimate partner violence**

- Enduring IPV because no options / for children
- Justifying violence because of a woman's duty
- Marital rape
- Emotional abuse
- Physical violence
  - Normalizing physical violence
- Reactions/responses to violence
  - How women cope with violence
- Reasons for Violence

**Sexual violence**

**Verbal abuse**

- Words hurt more than hitting

**Different experiences based on social class in Syria**

- Racism toward other Syrians of different backgrounds

**Forced child labour in Lebanon**

- Risk of sexual violence against children

**Sex work**

- Drivers of sex work

**Sexual harassment**

**Taking advantage**

- Trafficking

**Kidnapping**

**Syrian cultural values / customs / traditions**

**Women are weak**

**Barriers to disclosure and care-seeking**

- Guilt
  - Guilt for coming to Lebanon
  - Guilt for having children
- Not discussing sex work
- Reactions to sex work
- Violation of "honor"
  - Keeping one's honor
  - Victim blaming
- Women can't speak to others
  - Fear of being blamed
  - No one to confide in
  - Unable to discuss sexual violence

**Household crowding/living with other families on the way to Lebanon**

**Extreme poverty**

- Child Education
- Economic needs in Lebanon
  - Food insecurity
  - High Cost of living
  - High costs of healthcare
- In debt
- Unable to afford education
- Unable to secure essential needs
- Living conditions in Lebanon
- Negative coping strategies
- Positive coping strategies
  - Receiving aid
- Shelter insecurity
  - Sharing a house
- Unable to find jobs

**Final Code Tree**

**Sexually transmitted infections**

- STI symptoms
- STIs leading to infertility/stillbirth
- Husband refusing STI evaluation or treatment
  - STI reinfection
  - Sequelae of advanced/untreated STIs
- Receiving STI treatment

**Social isolation**

- Family separation
- Wanting to return to Syria
  - Unable to return to Syria because men forced to join group
  - Nothing to return to in Syria
- Women keeping to themselves and not interacting with society
  - Restricting women’s mobility in Lebanon
    - Dependence on men for safety
    - Lebanon is like a prison

**Internalized stigma and guilt**

- Guilt for coming to Lebanon
- Guilt for having children
- Fear of being blamed
- No one to confide in
  - Unable to discuss sexual violence
  - Not discussing STIs

**Intimate partner violence**

- Emotional violence
  - Words hurt more than hitting
- Sexual violence
  - Marital rape
  - Early / forced marriage
- Physical violence

**Sexual harassment and transactional sex**

- Examples of sex work / harassment
  - Shop-owners
  - Employers
- Women describing sex work among others
- Drivers of sex work

**Sexual exploitation and trafficking**

- Using aid/assistance as a guise for sexual violence
  - Trafficking / kidnapping
  - Rape

**Poor care accessibility**

- Labour and delivery
- Unable to see doctor
  - Self-treatment

**Patriarchal gender norms and stigma**

- Normalizing/justifying violence
  - Women’s obligations to spouses
- Syrian cultural values / customs / traditions
- Men are strong / women are weak
- Economic / social dependence on violent intimate partner
  - Enduring IPV for children / nowhere to go
- Shaming women engaging in sex work
  - Violation of "honor"

**Extreme poverty and insecurity**

- Women responsible for family in Lebanon
- Unable to afford children’s tuition
- Food/shelter insecurity
  - Household crowding
- Coping strategies
  - Child labour
  - Borrowing money
- Poverty driving IPV
